# Severe pneumonia induces immunosenescence of T cells in the lung of mice

**DOI:** 10.18632/aging.204893

**Published:** 2023-07-24

**Authors:** Qingle Ma, Chenhui Weng, Chenlu Yao, Jialu Xu, Bo Tian, Yi Wu, Heng Wang, Qianyu Yang, Huaxing Dai, Yue Zhang, Fang Xu, Xiaolin Shi, Chao Wang

**Affiliations:** 1 Laboratory for Biomaterial and Immunoengineering, Institute of Functional Nano and Soft Materials (FUNSOM), Soochow University, Suzhou 215123, Jiangsu, China; 2 Medical College of Soochow University, Suzhou 215123, Jiangsu, China; 3 Department of Orthopedics, The Second Affiliated Hospital of Soochow University, Suzhou 215004, Jiangsu, China

**Keywords:** pneumonia, naïve T, immunosenescence, aging, cancer

## Abstract

Severe pneumonia may induce sequelae and accelerated aging process even after the person has recovered. However, the underline mechanism is not very clear. More research is needed to fully understand the long-term effects of severe pneumonia. In this study, we found that mice recovered from severe pneumonia showed lung immunosenescence, which was characterized by a bias naive-memory balance of T lymphocytes in the lung. The reduction of naïve T cells is associated with the diminished immune response to cancer or external new antigens, which is one of the key changes that occurs with age. Our results also indicate the link between severe pneumonia and aging process, which is mediated by the disrupted T cells homeostasis in the lungs after pneumonia.

## INTRODUCTION

Severe pneumonia is a type of lung infection that is characterized by inflammation and fluid buildup in the air sacs of the lungs. It is a serious condition that can lead to respiratory failure and death. For example, the COVID-19 associated pneumonia caused by the novel coronavirus, SARS-CoV-2, has caused enormous damage to global health [1, 2]. Additionally, some people may experience ongoing fatigue and weakness after recovering from severe pneumonia, which can contribute to accelerated aging [3, 4]. Severe pneumonia can cause damage to the lungs, which may result in long-term lung sequelae even after the person has recovered [5–7]. A disrupted immunity homeostasis in the lungs can occur following the severe pneumonia [8, 9]. However, the link between aging and perturbed immune homeostasis in lung tissues remains unclear. More research is needed to fully understand the long-term effects of severe pneumonia.

During aging, the decline in immune function is manifested by the immune system losing its effective response to pathogens and cancer cells, a phenomenon known as immunosenescence [10]. T cell aging plays a major role in body-wide deterioration. There is abundant evidence in mice indicating that the physiological function of T lymphocytes is gradually compromised with age, leading to significant age-dependent changes. One of the key changes that occurs with age is a decline in the naïve T cell pool, which means that older individuals have fewer naïve T cells available for the immune system to continuously respond to unfamiliar pathogens such as cancer or external antigens [11, 12]. This is strongly associated with the increased propensity to develop autoimmune, autoinflammatory, infectious, and malignant diseases in aging of an individual. Therefore, the aging of T cells may be considered as a major expression of “immunosenescence,” which refers to the gradual decline in the immune system’s effectiveness over time.

Most study on the naive–memory balance of T lymphocytes in humans is largely limited to the peripheral blood, lymph nodes (LN), or gut [13, 14]. There is limited research on investigating the lung naive–memory balance of T lymphocytes after pneumonia. In this study, we found that mice recovered from severe pneumonia showed lung immunosenescence, which was characterized by decreased pulmonary naïve T cells and increased memory T cells, leading to a decline immune response to cancer or external antigens. It is found that the immune landscape of the lungs in mice recovered from severe pneumonia was significantly reshaped, which was characterized by a significant decline in the frequency of naïve T cells (CD62L+CD44-) along with the accumulation of highly differentiated memory T cells (CD62L-CD44+). In addition, we found the lung tissue, especially the lung T cells, expressed the senescence-associated secretory phenotype (SASP) after recovery from severe pneumonia. We further observed that recovered mice showed a low response to cancer or external antigens compared to the naïve mice due to the loss of naïve T cell pool. Our results indicate that there is an immunosenescence in the lungs after severe pneumonia, which may weaken immune system to response to cancer or external new antigens.

## RESULTS

### A disrupted immune landscape in the lung of mice recovered from severe pneumonia

**Figure 1:**
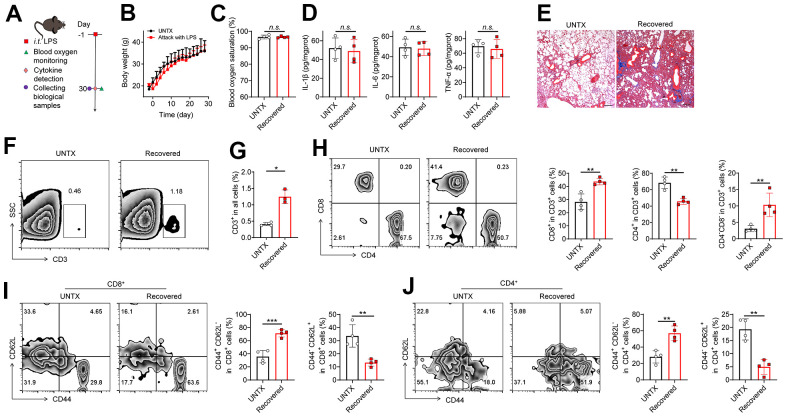
**A disrupted immune landscape in the lung of mice recovered from severe pneumonia.** (**A**) Schematic of the experimental timeline. Untreated (UNTX, mice not treated with LPS) and LPS-treated mice were then monitored for blood oxygen, and samples were collected for molecular pathological assessment. (**B**) Body weight of untreated and LPS-treated mice. (**C**) Blood oxygen saturation data of untreated and LPS-treated mice. (**D**) Inflammatory factors, including IL-1β, IL-6, and TNF-α, in lung tissue homogenate. (**E**) Representative Masson staining of lung sections after various treatments as indicated. Scale bars, 200 μm. (**F**) Representative plots of CD3^+^ cells as a percentage of the total cell population in lung tissues and (**G**) corresponding quantification results. (**H**) Representative flow cytometric analysis of T cells and quantitation of the percentage of CD8^+^ and CD4^+^ cells among CD3^+^ T cells in lung tissues. (**I**) Representative flow cytometry chart of naïve and memory CD8^+^ T cells in lung tissues and corresponding quantification results. (**J**) Representative flow cytometry chart of naïve and memory CD4^+^ T cells in lung tissues and corresponding quantification results. Data are shown as the mean ± SD (n=4). Statistical significance was calculated by Student’s t test (two-tailed) and one-way ANOVA using the Tukey posttest. *P < 0.05; **P < 0.01; ***P < 0.001; n.s. nonsignificant.

We established LPS-induced pneumonia (in tracheal injection) to study the changes in the immune context of mice after the pneumonia subsided (Figure 1A). The result showed that the mice experienced temporary weight loss after LPS attack but returned to normal at day 30 (Figure 1B). The levels of blood oxygen saturation and lung cytokines such as IL-1β, IL-6, and TNF-α were at normal range to those of untreated mice at 30 days (Figure 1C, 1D), indicating the respiratory function was recovered and inflammatory condition of the lung was regressed after 30 days. However, in line with previous study [15], pulmonary fibrosis progression was observed in lung tissues after the pneumonia subsided, as shown by Masson staining (Figure 1E), indicating the lungs undergo a healing process in which scar tissue forms.

The role of T cells during aging is receiving more attention due to their significant impact on overall immune responses [11, 12]. Next, we analyzed the T cell frequency and phenotype in the lungs of mice recovered from pneumonia at day 30 (Figure 1F–1J, Supplementary Figure 1). Naïve mice were used as controls. We observed that the frequency of total lung-infiltrating T cells (CD3^+^) increased about twofold higher in mice recovered from pneumonia (Figure 1F, 1G). Meanwhile, the frequency of CD8^+^ T cells and CD4^-^CD8^-^ T cells elevated, while CD4^+^ T cells decreased obviously in the lungs of mice after pneumonia (Figure 1H). The expansion of CD3^+^ T cells is consistent with previous studies showing that the CD3^+^ T cells actively participate in modulating the innate host responses to murine pulmonary infection and contribute to protective immunity during pneumonia recovery in adaptive immune response [16–18]. The strong response of CD8^+^ T-cell subsets contributes to the development of chronic pulmonary sequelae in elderly individuals after they are cured of acute pneumonia [19]. CD4^-^CD8^-^ T cells were also reported a protective role after ischemia–reperfusion injury (IRI) of lungs [20]. In addition, two adhesion molecules CD62L (L-selectin) and CD44 (H-CAM) have been adopted as markers differentiating naive and memory T cells [21]. Notably, we discovered that there is a striking increase in the frequency of memory T cells (CD44^+^CD62L^-^) while a dramatic decrease was observed in the population of naive T cells (CD44^-^CD62L^+^) in both CD4^+^ and CD8^+^ T cells of mice recovered from pneumonia compared to control mice (Figure 1I, 1J). Naive T cells are a type of T lymphocyte that has not yet encountered its specific antigen while memory T cells are specialized T cells that carry out specific functions in the immune response against an antigen [22]. With age, the naïve pool contracts along with the accumulation of memory T cells [22]. Our results suggest T cell immunosenescence in the lungs of mice recovered from pneumonia.

### Senescence of the lungs after severe pneumonia

**Figure 2:**
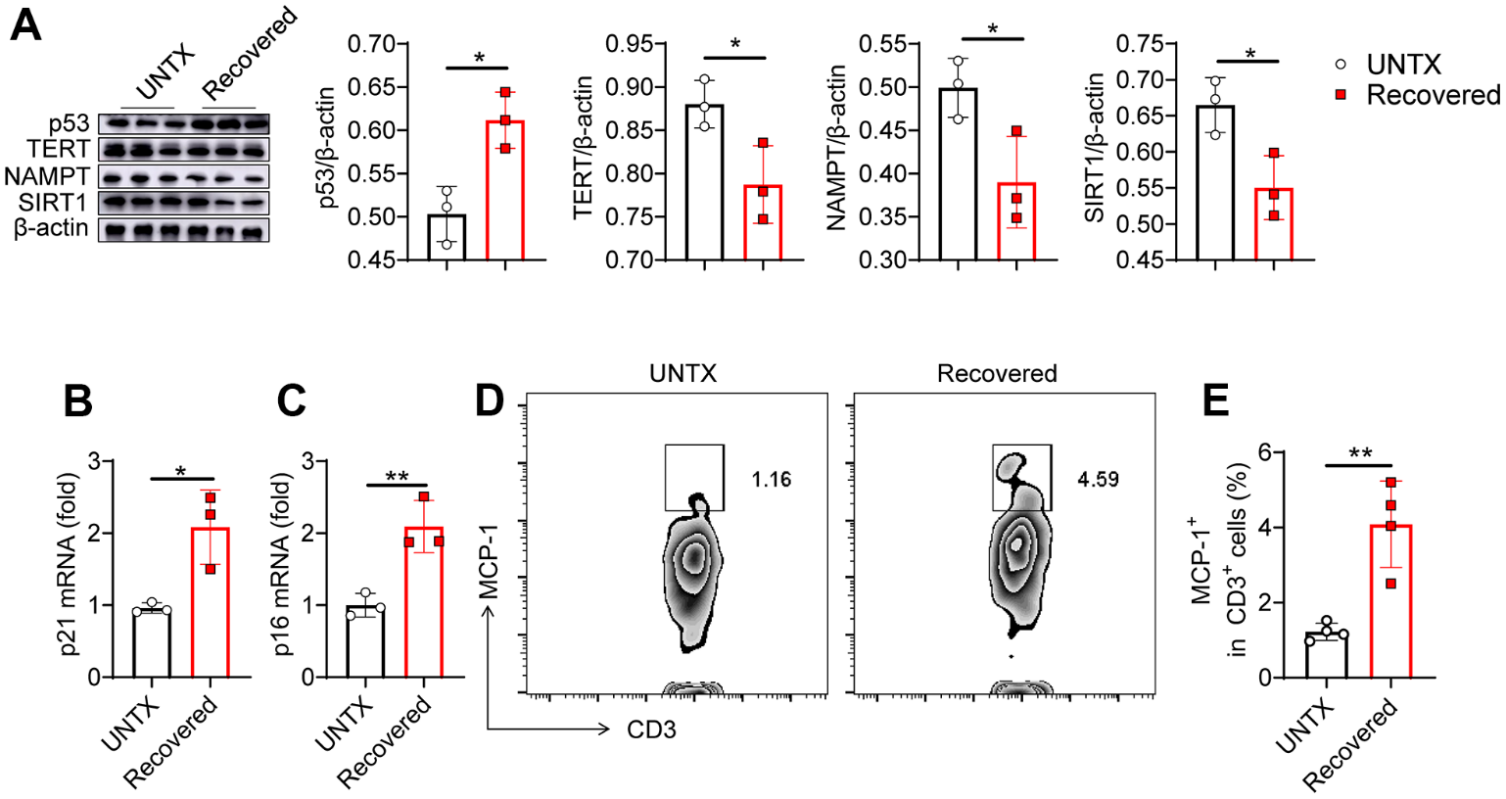
**Senescence of the lungs after severe pneumonia.** (**A**) Western blot analysis of the expression of various types of proteins in lung tissues after various treatments as indicated and the relative expression of proteins compared to the untreated group (n=3). (**B**, **C**) p21 and p16 mRNA expression analysis by qPCR (n=3). (**D**) Flow cytometric analysis for MCP-1^+^ in CD3^+^ cells of lung tissue and (**E**) corresponding quantification results (n=4). Data are shown as the mean ± SD. Statistical significance was calculated by Student’s t test (two-tailed) and one-way ANOVA using the Tukey posttest. *P < 0.05; **P< 0.01.

To further validate the immunosenescence of the lungs after severe pneumonia, we investigated several biologic hallmarks of aging in the lung tissue by various approaches. p53 activation can cause cell growth arrest or cell apoptosis and senescence, which is related to telomere dysfunction [23]. Telomerase reverse transcriptase (TERT) overexpression can stabilize telomeres and delay aging [24]. Niacinamide phosphoribosyl transferase (NAMPT) is mainly used to produce β-niacinamide mononucleotides *in vivo*, which are further converted to NAD^+^ [25]. SIRT1 protein levels decrease gradually with the aging process [26]. In our experiment, using western bolt assay, increased expression of p53 and decreased expression of TERT, NAMPT and SIRT1 were detected in the lung tissue of mice recovered from pneumonia, indicating that severe pneumonia accelerates lung aging (Figure 2A). We next detected the expression of mRNA p21 and p16 in the lung tissues by qPCR. They are both genes involved in the regulation of cell cycle, which have been linked to the aging process, with their expression increasing as cells age [27]. We found that both p21 and p16 mRNA expression were significantly increased in the lung tissues of recovered mice compared to that in untreated mice (Figure 2B, 2C). In addition, the level of monocyte chemoattractant protein-1 (MCP-1) increases with age, which is a hallmark of many age-related diseases [27]. Flow cytometry analysis of the expression of MCP-1 on CD3^+^ T cells showed that the T cells in lung tissue and peripheral blood also showed an aging pattern (Figure 2D, 2E and Supplementary Figures 2, 3). All these data indicated that pneumonia increased features of senescence in the lung, leading to a senescence-like phenotype of cellular senescence including T cells. Previous study showed that senescent cells appear in the lungs within hours following injury [28, 29]. Similarly, the rapid appearance of senescence markers was also reported in the liver in an acute injury model of hemorrhagic shock [28, 29], which was attributed to protection. Our result is consistent with recent reports, suggesting that senescence appearance following the injury may also contribute to the aging phenotype.

### Recovered mice showed a low anticancer response in the lung

Naive T cells can differentiate into tumor antigen-specific T effector cells that encounter new tumor antigen for the first time in the body. However, as the decline in number, diversity, and functionality of naive T, they compromise the response to new tumor antigens [11]. We next questioned whether the lung cancer was promoted in the recovered mice after severe pneumonia.

**Figure 3:**
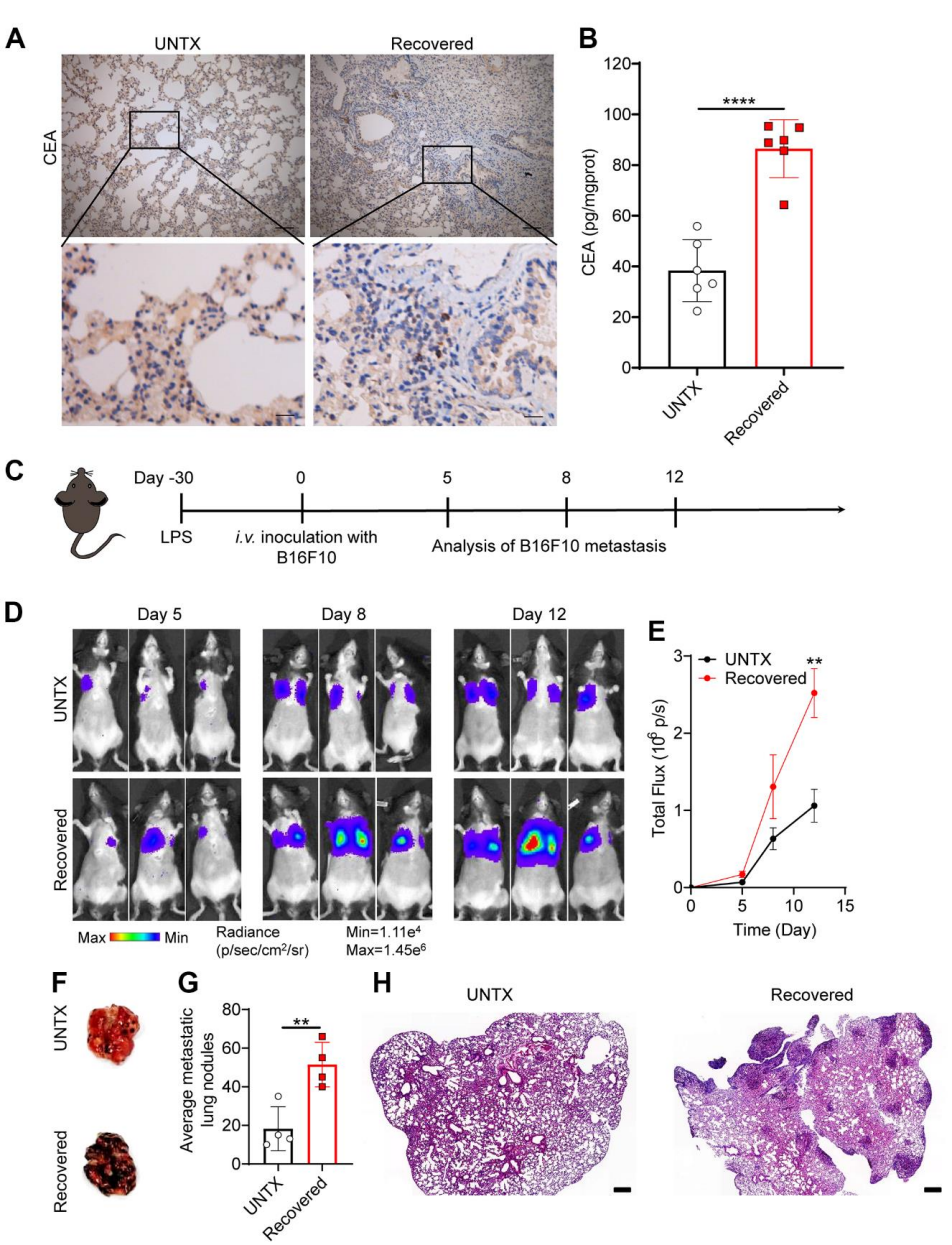
**Recovered mice showed a low anticancer response in the lung.** (**A**) Representative immunohistochemistry images of CEA-immunoreactive cells in lung sections of different groups. Scale bars, top, 100 μm; bottom, 20 μm. (**B**) Carcinoembryonic antigen (CEA) content in lung tissue homogenate. (**C**) Schematic of the experimental timeline. The tumor cells were then injected intravenously into untreated and 30D post-LPS treated mice to establish lung metastatic tumor model. (**D**) *In vivo* bioluminescence imaging of the B16F10-Luc lung metastasis tumor model in untreated and recovered groups and (**E**) corresponding quantification results. (**F**) Representative lung photographs, (**G**) number of lung tumor lesions, and (**H**) H&E-stained lung slices. Scale bars: 250 μm. Data are shown as the mean ± SD (n=4-6). Statistical significance was calculated by Student’s t test (two-tailed) and one-way ANOVA using the Tukey posttest. **P< 0.01; ****P < 0.0001.

Carcinoembryonic antigen (CEA) is a common tumor antigen marker that is often used in tumor screening and diagnosis [30]. In the clinical process, the increase in CEA is mainly seen in many cancers including lung cancer [31]. In addition, elevated CEA has been found in aging and chronic obstructive pulmonary disease, which is related to oxidative stress and chronic low-grade inflammation [32, 33]. Interestingly, from immunohistochemical imaging, we discovered that CEA levels in the lung tissue of mice obviously increased at day 30 after LPS attack compared to controls, suggesting potential tumorigenesis in the lung (Figure 3A, 3B). To further investigate whether tumor colonization in the lungs was promoted in the recovered mice after severe pneumonia, we established an experimental mouse lung metastatic tumor model by intravenous injection of 2×10^5^ B16F10-Luc tumor cells on day 30 after LPS attack (Figure 3C). As expected, recovered mice showed an accelerated melanoma colonization and progress in the lungs compared to untreated mice (Figure 3D, 3E). Lung photographs of metastases and H&E staining further showed that there were significantly more tumor cells in the lung than in untreated mice (Figure 3F–3H). These data support the notion that recovered mice showed a low anticancer response in the lung due to the T cell aging that was inferior to response to tumor antigens.

### Recovered mice showed a low antigen response systemically

**Figure 4:**
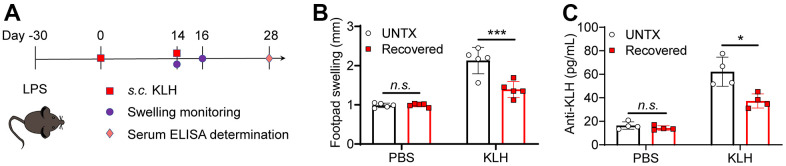
**Recovered mice showed a low antigen response systemically.** (**A**) Schematic of the experimental timeline. Untreated and recovered mice were immunized with KLH antigen, and the paw swelling was observed and serum ELISA was performed. (**B**) KLH delayed-type hypersensitivity data after sensitization of untreated and recovered mice (n=5). Footpad swelling at 48 h after challenge. (**C**) KLH antibodies measured by ELISA one month after challenge (n=4). Data are shown as the mean ± SD. Statistical significance was calculated by Student’s t test (two-tailed) and one-way ANOVA using the Tukey posttest. *P < 0.05; **P< 0.01; ***P < 0.001.

Elder people usually have a low vaccine response rate due to the decline of naive T cells [34]. In addition to the lung tissue, we next explore antigen response systemically in recovered mice. Delayed-type hypersensitivity (DTH) tests were performed to assess cell-mediated immunity in response to an antigen (Figure 4B). Keyhole limpet hemocyanin (KLH) is a xenogeneic protein antigen that can effectively trigger immune response in mice [35]. The recovered mice were immunized with KLH by footpad twice. The naïve mice were used as control. Similarly, diminished pad swelling and serum anti-KLH antibody levels were observed in mice recovered from pneumonia (Figure 4B, 4C). This result indicated the recovered mice had a low antigen response systemically.

## DISCUSSION

Severe pneumonia can have lasting effects on the body, including the potential for accelerated aging. Previous studies suggested that this may be due to the damage caused by inflammation and oxidative stress in the body during the infection [36, 37]. It also has the impact on the immune system and other bodily processes. More research is needed to fully understand the long-term effects of severe pneumonia.

Naive T cells are a subset of T lymphocytes that have not been exposed to antigen. A reduction in the production of naive T cells hinders the ability to recognize antigens. The number of distinct antigens that T cells can recognize is directly related to the number of clones within the naive T cell population. As individuals age, the naive pool decreases, while the number of highly differentiated memory cells increases. This shift towards a memory phenotype can be attributed to the depletion of stem cell-like pools within the T cell lineage, thereby weakening the response to new antigens [11].

Qin et al. reported the decreased naïve CD4^+^ and CD8^+^ T cells, increased memory CD4^+^ or CD8^+^ T cells were significant for aging of immune system in 1068 Chinese healthy volunteers ranging from 18 to 80 years old [38]. Studies also point to an age-associated decrease in naive T cells in the gut-associated lymphoid tissue [39], as well as in lymph nodes and the spleen [40]. However, there is limited research investigating the lung naive–memory balance of T lymphocytes after pneumonia. In this study, we found that the aging of T cells can be induced by pneumonia. Severe pneumonia could significantly reshape the T cell phenotype in the lung, characterized by a striking reduction of naïve T cells along with an accumulation of the memory T cells phenotype.

Generally, the older people show low response to vaccine [41], and cancer risk increases exponentially with age [42], while the aging of T cells is likely to play an essential role in this process. Age-related decline in number, diversity, and functionality of naive T cells and the bias towards the memory phenotype compromises the response to new antigens including tumor antigens and vaccine antigens. In this work, we further found that severe pneumonia was associated with an increased risk of developing lung cancer by inducing T cells aging in the lung tissue. In addition to the lung, the host showed a low response to xenogeneic antigens after recovered from server pneumonia. The above observations are indicative of the scenario that severe pneumonia induced the aging of T cells, resulting the diminished immune response to cancer or external new antigens.

## CONCLUSIONS

In summary, we investigated the link between aging and server pneumonia in mice. We found that mice recovered from severe pneumonia showed lung immunosenescence, which was characterized by decreased pulmonary naïve T cells and increased memory T cells, leading to a decline immune response to cancer or external antigens. We delineated bias lung naive–memory balance of T lymphocytes after severe pneumonia, indicating immunosenescence in the lungs after severe pneumonia, which may weaken immune system to response to lung cancer or external new antigens.

## MATERIALS AND METHODS

### Materials

The following materials were used in this study.

**Table 1:** Reagent used in this study.

**REAGENT or RESOURCE**	**SOURCE**	**IDENTIFIER**
**Antibodies**		
FITC anti-mouse CD3	Biolegend	Cat # 100204
APC anti-mouse CD4	Biolegend	Cat # 100412
PE anti-mouse CD8a	Biolegend	Cat # 100708
PE anti-mouse CD45	Biolegend	Cat # 103106
PE anti-mouse CD44	Biolegend	Cat # 103008
APC anti-mouse CD62L	Biolegend	Cat # 104412
PE anti-mouse FOXP3	Biolegend	Cat # 126403
APC anti-mouse IL-4	Biolegend	Cat # 504105
PE anti-mouse/rat/human MCP-1	Biolegend	Cat # 505903
Anti-CEA	Servicebio	Cat # GB112292
Anti-p53	Abmart	Cat # TA0879
Anti-TERT	Abmart	Cat # TD7129
Anti-NAMPT	Abmart	Cat # TD6059
Anti-SIRT1	Abmart	Cat # TD6033
Anti-β-actin	Servicebio	Cat # GB11001
**Drug**		
LPS	Biosharp	Cat # BS904
Freund’s complete adjuvant	Biosharp	Cat # BS156
Keyhole limpet	Yuanye	Cat # S25146
**Critical Commercial Assays**		
BCA Protein Assay Kit	Beyotime	Cat # P0012
PAGE Gel Rapid Preparation Kit	Epizyme	Cat # PG113
Mouse IL-1 beta Uncoated ELISA Kit	Invitrogen	Cat # 88-7013-88
Mouse IL-6 Uncoated ELISA Kit	Invitrogen	Cat # 88-7064-88
Mouse TNF alpha Uncoated ELISA Kit	Invitrogen	Cat # 88-7324-88
Ultrapure RNA Kit	CoWin Biosciences	Cat # CW0581S
HiFiScript gDNA Removal cDNA	CoWin Biosciences	Cat # CW2582M
SYBR qPCR SuperMix plus	Novoprotein	Cat # E096-01A
**Oligonucleotides**		
P21 F1: GTCAGGCTGGTCTGCCTCCG	Genewiz	N/A
P21 R1: CGGTCCCGTGGACAGTGAGCAG	Genewiz	N/A
P16 F1: CCCAACGCCCCGAACT	Genewiz	N/A
P16 R1: GCAGAAGACTGCTACGTGAA	Genewiz	N/A
GAPDH F1: AAGGTCATCCCAGAGCTGAA	Genewiz	N/A
GAPDH R1: CTGCTTCACCACCTTCTTGA	Genewiz	N/A

### Animals

C57BL/6 mice aged 8 weeks (half male and half female) were purchased from Nanjing Peng Sheng Biological Technology Co., Ltd. There was an at least 7-day gap between the time of purchasing mice and our experiment on them to ensure that they were accustomed to the conditions of the laboratory. The mice were housed in a vivarium maintained at 20 ± 2° C and 55% humidity with a 12-h light–dark cycle and free access to food and water. The housing group was five at maximum for mice in each group.

We used the ARRIVE reporting guidelines. The aim of this study was to investigate the pulmonary immune microenvironment after recovery from severe pneumonia. Animal experiments shall be approved and supervised by the Animal Welfare Review Committee after the purpose, method and ethics of the experiments are clarified. Mice were randomly divided into groups. No animals were excluded from the study, and the researchers conducted the experiments independently and evaluated the results. The mice were euthanized at the end of the experiment or when they had health problems. All experiments were repeated at least 3 times.

### Animal model induction

For LPS treatment, healthy mice were anaesthetized with isoflurane. We placed each mouse in an air-numbed chamber and adjusted the oxygen flowmeter to between 0.6 and 1.2 L/min. Once fully anaesthetized, the mice were fixed in the supine position. The mouth of the mice was opened, the tongue was picked out with forceps and placed in the lateral position, and the exposed tracheal hole was observed under a spotlight. LPS (4 mg/kg) (50 μL) was injected into the trachea through a syringe.

### Body weight and oxygen saturation measurements

LPS-challenged mice and untreated healthy mice as controls were weighed at the same time and monitored every two days until 30 days after modelling. In addition, blood oxygen saturation was measured by a MouseOx® (STARR) oxygen detector immediately after LPS attack. The mice were included in the study if the blood oxygen saturation dropped remarkably, and these mice were enrolled as the experimental group. Healthy mice of the same age without any treatment were utilized as controls, and their weight was monitored together with the experimental group.

### Cytokine detection

After the tissue samples were rinsed with precooled PBS, samples with the same weight were weighed and ground in a tissue grinder to make a 10% tissue homogenate. The prepared homogenate was centrifuged at 6000×g for 10 min. The supernatant of each homogenate sample was collected, and the total protein content of each tissue homogenate sample was determined by the BCA method. After that, the reaction was performed on the precoated enzyme-labelled plate, followed by washing, enzyme-labelled antibody coupling, substrate reaction, and termination of the reaction operation. Finally, the multifunctional enzyme-labelled instrument was used for detection at wavelengths of 450 nm and 570 nm.

### Western blotting

After the brain tissue of each experimental group was obtained, a mixed solution of radioimmunoprecipitation assay (RIPA) lysate and phenylmethanesulfonylfluoride (PMSF) (100:1) was added. After lysis in an ice bath, the protein supernatant was obtained by centrifugation at 12000 rpm, and the protein was quantified by a BCA protein quantification kit. The denatured protein was mixed with 5× load buffer and isolated by 12.5% SDS-PAGE at 60 V for 30 min and 120 V for 90 min. After that, the protein was transferred to a PVDF membrane in an ice bath for 100 min and then blocked in 5% skim milk powder solution for 1 h. After that, the protein was incubated with β-actin (1:2000, Serviceio), anti-p53 (1:1500, Abmart), anti-TERT (1:1500, Abmart), NAMPT (1:1500, Abmart), and SIRT1 (1:1500, Abmart) at 4° C overnight. After that, goat anti-rabbit secondary antibody (1:5000, Absin) coupled with horseradish peroxidase was incubated at room temperature for 1 hour, bands were displayed by chemiluminescence development, and data quantitative analysis was performed by the ImageJ software package.

### RNA isolation and qRT–PCR

Tissue was collected from euthanized mice and rapidly frozen in liquid nitrogen. Total RNA was extracted and separated by TRIzol. The purity and concentration of total RNA were determined by an ultra-micro nucleic acid protein analyzer (DeNovix, DS-11FX+). The first strand of cDNA was synthesized by reverse transcription according to the instructions of the reverse transcription kit and was amplified by quantitative fluorescent PCR with 3 reholes per sample. The amplification conditions were as follows: 95° C for 5 min, followed by 40 cycles of 95° C for 10 s, 60° C for 30 s, and 72° C for 5 s. The calculation method was Ratio = 2^-ΔΔCT^, ΔCT1 = CT value of target gene in control - CT value of reference gene in control, ΔCT2= CT value of target gene in experimental group - CT value of reference gene in experimental group, ΔΔCT= ΔCT2-ΔCT1. The generation formula 2^-ΔΔCT^ was used to calculate the relative mRNA content of the experimental group and the negative control group.

### Lung metastatic tumor model

The C57BL/6 mice were divided into two groups, including the UNTX and 30D post-LPS groups. At 30 days after LPS attack, 2×10^5^ B16F10-Luc cells were injected intravenously to construct a melanoma lung metastasis model. An *in vivo* fluorescence imaging system was used on days 5, 8 and 12 after tumor cell injection. The operation was set as 10 minutes after intraperitoneal injection of 10 μL/g d- luciferin potassium salt (15 mg/mL). Exposure time: 5 minutes.

### Histological analysis

After treatment, major organs of mice in each group were obtained, cleaned in PBS to remove excess blood, fixed in 4% paraformaldehyde solution, and then embedded in paraffin. The paraffin samples were cut into 4 μm thick slices for hematoxylin-eosin staining, Masson staining and CEA immunohistochemical staining. Then, the pathological status of the samples was observed and analyzed by optical microscopy.

### Flow cytometric immunoassay of mouse tissue

Peripheral blood and lung tissues of mice in the experimental groups were obtained. Erythrocyte lysate was added to peripheral blood to remove erythrocytes, and antibody staining was performed after washing and centrifugation with PBS. A tissue cell suspension was obtained using a tissue grinder. The single-cell suspension was then filtered through a filter, washed and centrifuged with PBS, and resuspended in FACS buffer solution (PBS containing 3% BSA). Furthermore, the cells were stained with anti-CD45-PE, anti-CD3-FITC, anti-MCP-1-PE, anti-CD8-PE, anti-CD4-APC, anti-FOXP3-PE, anti-IL4-APC, anti-CD44-PE, and anti-CD62L-APC (BioLegend). The stained cells were analyzed using a BD Accuri C6 flow cytometer and FlowJo V10 software based on 100,000 gated events. Fluorescence minus one (FMO) controls were used to set gates for determine the percentage of T cell subsets.

### Delayed-type hypersensitivity measurements

After anaesthetizing mice, 100 μL KLH antigen (2 mg mL^-1^) was injected subcutaneously into the back skin of mice emulsified 1:1 with Freund’s complete adjuvant. After 2 weeks of sensitization, the anaesthetized mice were again injected with 20 μg KLH into the rear foot pads. After the mice regained consciousness, they were sent back to the cage for monitoring. Posterior paw swelling was monitored with Vernier calipers at 0 and 48 hours after antigen administration.

### Anti-KLH antibody ELISA

Mouse orbital blood was collected for anti-KLH antibody detection. Similar to the cytokine assay, the reaction was performed on a precoated enzyme-labelled plate followed by washing, enzyme-labelled antibody coupling, substrate reaction, and termination of the reaction. Finally, multifunctional enzyme labelling instruments were used for detection at 450 nm and 570 nm.

### Statistical analysis

All data in the present study are means ± standard deviations. The significance of differences between two groups was calculated by a two-tailed unpaired Student’s t test. In addition, analysis of variance (ANOVA) comparisons and Tukey post hoc tests were performed between more than two groups (multiple comparisons). All statistical analyses were performed using GraphPrism (v5.0). Values of *P* = 0.05 or less were considered significant. All intensities of fluorescence expression in the experiments were further calculated by ImageJ software. The standard symbols are presented as *P < 0.05, **P< 0.01, ***P < 0.001, and ****P < 0.0001.

## Supplementary Material

Supplementary Figures
