# Supplementary material for: Severe pneumonia induces immunosenescence of T cells in the lung of mice
Source: Aging (Albany NY). 2023 Jul 24;15(14):7084–97. doi: 10.18632/aging.204893 (PMC10415552; doi:10.18632/aging.204893)
Supplement: Supplementary Figures [file aging-15-204893-s001.pdf]

SUPPLEMENTARY FIGURES

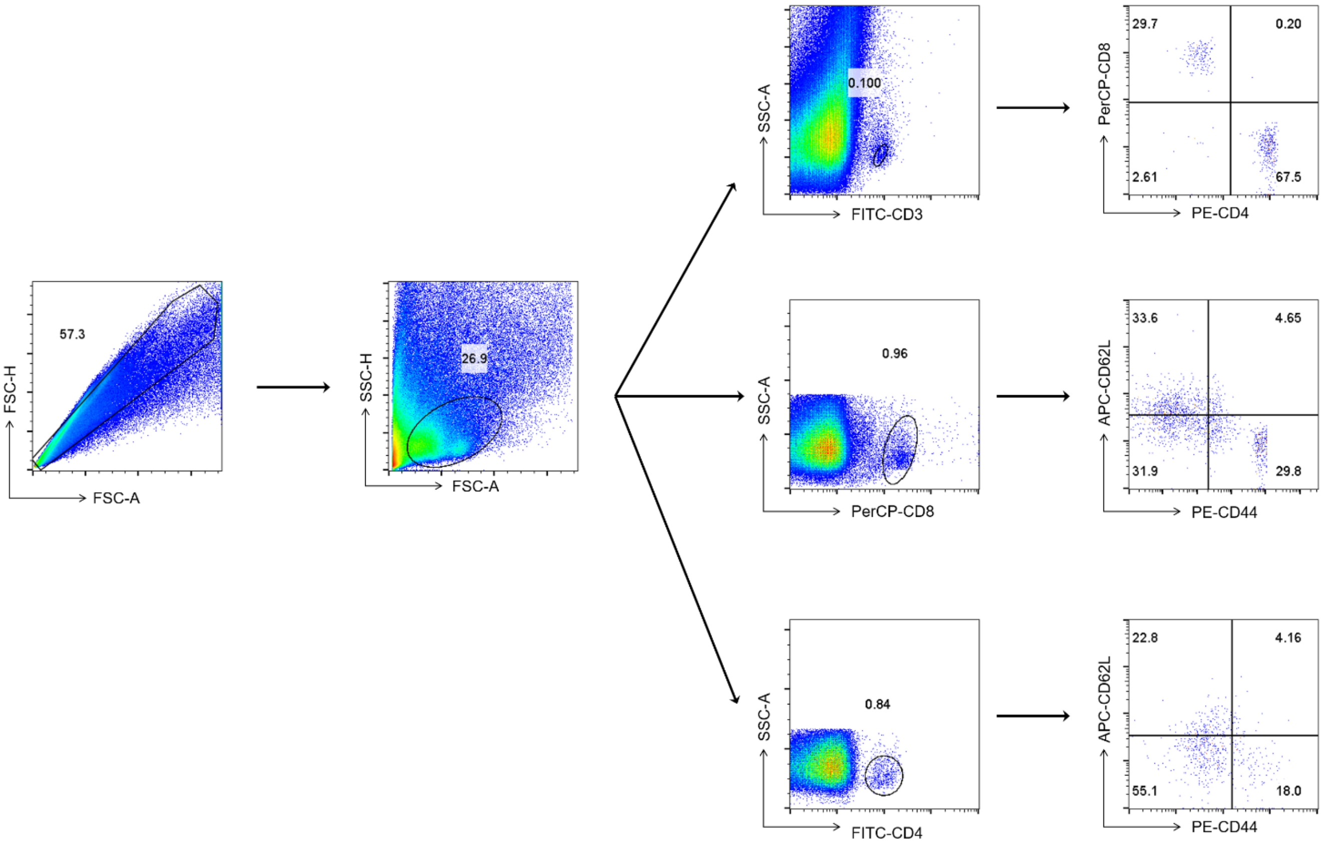

Supplementary Figure 1. (Related to Figure 1). Flow cytometry gating strategy for analysis of T cells subsets.

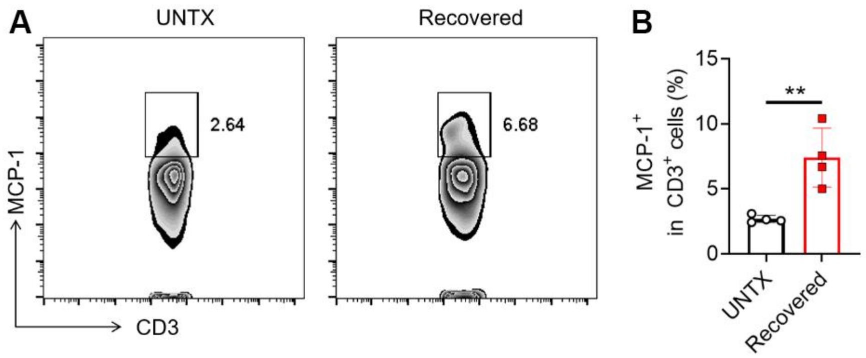

Supplementary Figure 2. (**A**) Flow cytometric analysis of MCP-1$^+$ in CD3$^+$ cells in peripheral blood and (**B**) corresponding quantification results (n=4). Data are shown as the mean ± SD. Statistical significance was calculated by Student's t test (two-tailed) and one-way ANOVA using the Tukey posttest. **P< 0.01.

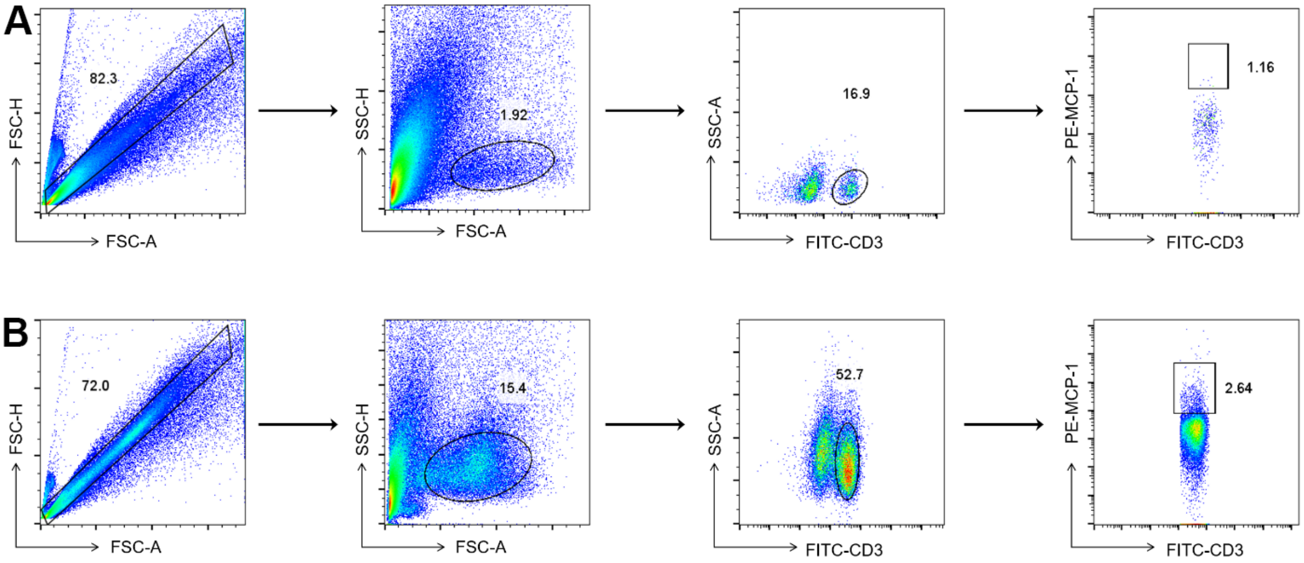

Supplementary Figure 3. (Related to Figure 2). Flow cytometry gating strategy for analysis of MCP-1$^+$ in CD3$^+$ cells. (**A**) Lung; (**B**) Peripheral blood.
